# Fostering Critical Thinking, Reasoning, and Argumentation Skills through Bioethics Education

**DOI:** 10.1371/journal.pone.0036791

**Published:** 2012-05-11

**Authors:** Jeanne Ting Chowning, Joan Carlton Griswold, Dina N. Kovarik, Laura J. Collins

**Affiliations:** 1 Northwest Association for Biomedical Research, Seattle, Washington, United States of America; 2 Center for Research and Learning, Snohomish, Washington, United States of America; University of South Alabama, United States of America

## Abstract

Developing a position on a socio-scientific issue and defending it using a well-reasoned justification involves complex cognitive skills that are challenging to both teach and assess. Our work centers on instructional strategies for fostering critical thinking skills in high school students using bioethical case studies, decision-making frameworks, and structured analysis tools to scaffold student argumentation. In this study, we examined the effects of our teacher professional development and curricular materials on the ability of high school students to analyze a bioethical case study and develop a strong position. We focused on student ability to identify an ethical question, consider stakeholders and their values, incorporate relevant scientific facts and content, address ethical principles, and consider the strengths and weaknesses of alternate solutions. 431 students and 12 teachers participated in a research study using teacher cohorts for comparison purposes. The first cohort received professional development and used the curriculum with their students; the second did not receive professional development until after their participation in the study and did not use the curriculum. In order to assess the acquisition of higher-order justification skills, students were asked to analyze a case study and develop a well-reasoned written position. We evaluated statements using a scoring rubric and found highly significant differences (p<0.001) between students exposed to the curriculum strategies and those who were not. Students also showed highly significant gains (p<0.001) in self-reported interest in science content, ability to analyze socio-scientific issues, awareness of ethical issues, ability to listen to and discuss viewpoints different from their own, and understanding of the relationship between science and society. Our results demonstrate that incorporating ethical dilemmas into the classroom is one strategy for increasing student motivation and engagement with science content, while promoting reasoning and justification skills that help prepare an informed citizenry.

## Introduction

While the practice of argumentation is a cornerstone of the scientific process, students at the secondary level have few opportunities to engage in it [1]. Recent research suggests that collaborative discourse and critical dialogue focused on student claims and justifications can increase student reasoning abilities and conceptual understanding, and that strategies are needed to promote such practices in secondary science classrooms [2]. In particular, students need structured opportunities to develop arguments and discuss them with their peers. In scientific argument, the data, claims and warrants (that relate claims to data) are strictly concerned with scientific data; in a socio-scientific argument, students must consider stakeholder perspectives and ethical principles and ideas, in addition to relevant scientific background. Regardless of whether the arguments that students employ point towards scientific or socio-scientific issues, the overall processes students use in order to develop justifications rely on a model that conceptualizes arguments as claims to knowledge [3].

Prior research in informal student reasoning and socio-scientific issues also indicates that most learners are not able to formulate high-quality arguments (as defined by the ability to articulate justifications for claims and to rebut contrary positions), and highlights the challenges related to promoting argumentation skills. Research suggests that students need experience and practice justifying their claims, recognizing and addressing counter-arguments, and learning about elements that contribute to a strong justification [4], [5].

Proponents of Socio-scientific Issues (SSI) education stress that the intellectual development of students in ethical reasoning is necessary to promote understanding of the relationship between science and society [4], [6]. The SSI approach emphasizes three important principles: (a) because science literacy should be a goal for all students, science education should be broad-based and geared beyond imparting relevant content knowledge to future scientists; (b) science learning should involve students in thinking about the kinds of real-world experiences that they might encounter in their lives; and (c) when teaching about real-world issues, science teachers should aim to include contextual elements that are beyond traditional science content. Sadler and Zeidler, who advocate a SSI perspective, note that “people do not live their lives according to disciplinary boundaries, and students approach socio-scientific issues with diverse perspectives that integrate science and other considerations” [7].

Standards for science literacy emphasize not only the importance of scientific content and processes, but also the need for students to learn about science that is contextualized in real-world situations that involve personal and community decision-making [7]–[10]. The National Board for Professional Teaching Standards stresses that students need “regular exposure to the human contexts of science [and] examples of ethical dilemmas, both current and past, that surround particular scientific activities, discoveries, and technologies” [11]. Teachers are mandated by national science standards and professional teaching standards to address the social dimensions of science, and are encouraged to provide students with the tools necessary to engage in analyzing bioethical issues; yet they rarely receive training in methods to foster such discussions with students.

The Northwest Association for Biomedical Research (NWABR), a non-profit organization that advances the understanding and support of biomedical research, has been engaging students and teachers in bringing the discussion of ethical issues in science into the classroom since 2000 [12]. The mission of NWABR is to promote an understanding of biomedical research and its ethical conduct through dialogue and education. The sixty research institutions that constitute our members include academia, industry, non-profit research organizations, research hospitals, professional societies, and volunteer health organizations. NWABR connects the scientific and education communities across the Northwestern United States and helps the public understand the vital role of research in promoting better health outcomes. We have focused on providing teachers with both resources to foster student reasoning skills (such as activities in which students practice evaluating arguments using criteria for strong justifications), as well as pedagogical strategies for fostering collaborative discussion [13]–[15]. Our work draws upon socio-scientific elements of functional scientific literacy identified by Zeidler et al. [6]. We include support for teachers in discourse issues, nature of science issues, case-based issues, and cultural issues – which all contribute to cognitive and moral development and promote functional scientific literacy. Our Collaborations to Understand Research and Ethics (CURE) program, funded by a Science Education Partnership Award from the National Institutes of Health (NIH), promotes understanding of translational biomedical research as well as the ethical considerations such research raises.

Many teachers find a principles-based approach most manageable for introducing ethical considerations. The principles include respect for persons (respecting the inherent worth of an individual and his or her autonomy), beneficence/nonmaleficence (maximizing benefits/minimizing harms), and justice (distributing benefits/burdens equitably across a group of individuals). These principles, which are articulated in the Belmont Report [16] in relation to research with human participants (and which are clarified and defended by Beauchamp and Childress [17]), represent familiar concepts and are widely used. In our professional development workshops and in our support resources, we also introduce teachers to care, feminist, virtue, deontological and consequentialist ethics. Once teachers become familiar with principles, they often augment their teaching by incorporating these additional ethical approaches.

The Bioethics 101 materials that were the focus of our study were developed in conjunction with teachers, ethicists, and scientists. The curriculum contains a series of five classroom lessons and a culminating assessment [18] and is described in more detail in the Program Description below. For many years, teachers have shared with us the dramatic impacts that the teaching of bioethics can have on their students; this research study was designed to investigate the relationship between explicit instruction in bioethical reasoning and resulting student outcomes. In this study, teacher cohorts and student pre/post tests were used to investigate whether CURE professional development and the Bioethics 101 curriculum materials made a significant difference in high school students’ abilities to analyze a case study and justify their positions. Our research strongly indicates that such reasoning approaches can be taught to high school students and can significantly improve their ability to develop well-reasoned justifications to bioethical dilemmas. In addition, student self-reports provide additional evidence of the extent to which bioethics instruction impacted their attitudes and perceptions and increased student motivation and engagement with science content.

## Methods

### Program Description

Our professional development program, Ethics in the Science Classroom, spanned two weeks. The first week, a residential program at the University of Washington (UW) Pack Forest Conference Center, focused on our Bioethics 101 curriculum, which is summarized in Table S1 and is freely available at http://www.nwabr.org. The curriculum, a series of five classroom lessons and a culminating assessment, was implemented by all teachers who were part of our CURE treatment group. The lessons explore the following topics: (a) characteristics of an ethical question; (b) bioethical principles; (c) the relationship between science and ethics and the roles of objectivity/subjectivity and evidence in each; (d) analysis of a case study (including identifying an ethical question, determining relevant facts, identifying stakeholders and their concerns and values, and evaluating options); and (e) development of a well-reasoned justification for a position.

Additionally, the first week focused on effective teaching methods for incorporating ethical issues into science classrooms. We shared specific pedagogical strategies for helping teachers manage classroom discussion, such as asking students to consider the concerns and values of individuals involved in the case while in small single and mixed stakeholder groups. We also provided participants with background knowledge in biomedical research and ethics. Presentations from colleagues affiliated with the NIH Clinical and Translational Science Award program, from the Department of Bioethics and Humanities at the UW, and from NWABR member institutions helped participants develop a broad appreciation for the process of biomedical research and the ethical issues that arise as a consequence of that research. Topics included clinical trials, animal models of disease, regulation of research, and ethical foundations of research. Participants also developed materials directly relevant and applicable to their own classrooms, and shared them with other educators. Teachers wrote case studies and then used ethical frameworks to analyze the main arguments surrounding the case, thereby gaining experience in bioethical analysis. Teachers also developed Action Plans to outline their plans for implementation.

The second week provided teachers with first-hand experiences in NWABR research institutions. Teachers visited research centers such as the Tumor Vaccine Group and Clinical Research Center at the UW. They also had the opportunity to visit several of the following institutions: Amgen, Benaroya Research Institute, Fred Hutchinson Cancer Research Center, Infectious Disease Research Institute, Institute for Stem Cells and Regenerative Medicine at the UW, Pacific Northwest Diabetes Research Institute, Puget Sound Blood Center, HIV Vaccine Trials Network, and Washington National Primate Research Center. Teachers found these experiences in research facilities extremely valuable in helping make concrete the concepts and processes detailed in the first week of the program.

We held two follow-up sessions during the school year to deepen our relationship with the teachers, promote a vibrant ethics in science education community, provide additional resources and support, and reflect on challenges in implementation of our materials. We also provided the opportunity for teachers to share their experiences with one another and to report on the most meaningful longer-term impacts from the program. Another feature of our CURE program was the school-year Institutional Review Board (IRB) and Institutional Animal Care and Use Committee (IACUC) follow-up sessions. Teachers chose to attend one of NWABR’s IRB or IACUC conferences, attend a meeting of a review board, or complete NIH online ethics training. Some teachers also visited the UW Embryonic Stem Cell Research Oversight Committee. CURE funding provided substitutes in order for teachers to be released during the workday. These opportunities further engaged teachers in understanding and appreciating the actual process of oversight for federally funded research.

### Participants

Most of the educators who have been through our intensive summer workshops teach secondary level science, but we have welcomed teachers at the college, community college, and even elementary levels. Our participants are primarily biology teachers; however, chemistry and physical science educators, health and career specialists, and social studies teachers have also used our strategies and materials with success.

The research design used teacher cohorts for comparison purposes and recruited teachers who expressed interest in participating in a CURE workshop in either the summer of 2009 or the summer of 2010. We assumed that all teachers who applied to the CURE workshop for either year would be similarly interested in ethics topics. Thus, Cohort 1 included teachers participating in CURE during the summer of 2009 (the treatment group). Their students received CURE instruction during the following 2009–2010 academic year. Cohort 2 (the comparison group) included teachers who were selected to participate in CURE during the summer of 2010. Their students received a semester of traditional classroom instruction in science during the 2009–2010 academic year. In order to track participation of different demographic groups, questions pertaining to race, ethnicity, and gender were also included in the post-tests.

Using an online sample size calculator http://www.surveysystem.com/sscalc.htm, a 95% Confidence Level, and a Confidence Interval of 5, it was calculated that a sample size of 278 students would be needed for the research study. For that reason, six Cohort 1 teachers were impartially chosen to be in the study. For the comparison group, the study design also required six teachers from Cohort 2. The external evaluator contacted all Cohort 2 teachers to explain the research study and obtain their consent, and successfully recruited six to participate.

### Ethics Statement

This study was conducted according to the principles expressed in the Declaration of Helsinki. Prior to the study, research processes and materials were reviewed and approved by the Western Institutional Review Board (WIRB Study #1103180). CURE staff and evaluators received written permission from parents to have their minor children participate in the Bioethics 101 curriculum, for the collection and subsequent analysis of students’ written responses to the assessment, and for permission to collect and analyze student interview responses. Teachers also provided written informed consent prior to study participation. All study participants and/or their legal guardians provided written informed consent for the collection and subsequent analysis of verbal and written responses.

### Research Study

#### Analyzing a case study: CURE and comparison students

Teacher cohorts and pre/post tests were used to investigate whether CURE professional development and curriculum materials made a significant difference in high school students’ abilities to analyze a case study and justify their positions. Cohort 1 teachers (N = 6) received CURE professional development and used the Bioethics 101 curriculum with their students (N = 323); Cohort 2 teachers (N = 6) did not receive professional development until after their participation in the study and did not use the curriculum with their students (N = 108). Cohort 2 students were given the test case study and questions, but with only traditional science instruction during the semester. Each Cohort was further divided into two groups (A and B). Students in Group A were asked to complete a pre-test prior to the case study, while students in Group B did not. All four student groups completed a post-test after analysis of the case study. This four-group model (Table 1) allowed us to assess: 1) the effect of CURE treatment relative to conventional education practices, 2) the effect of the pre-test relative to no pre-test, and 3) the interaction between the pre-test and CURE treatment condition. Random assignment of students to treatment and comparison groups was not possible; consequently we used existing intact classes. In all, 431 students and 12 teachers participated in the research study (Table 2).

**Table 1:** Four-Group Research Design.

Group	September	October–November	December
Cohort 1 – Treatment (CURE) Group A	Pre-test	Bioethics 101	Post-test
Cohort 1 – Treatment (CURE) Group B		Bioethics 101	Post-test
Cohort 2 – Comparison Group A	Pre-test		Post-test
Cohort 2 – Comparison Group B			Post-test

**Table 2:** Participants in the CURE Research Study.

Cohort 1 (CURE Treatment Group)				Cohort 2 (Comparison Group)			
Teacher	Group A (course)	Pre-test (N)	Post-test (N)	Teacher	Group A (course)	Pre-test (N)	Post-test (N)
**1**	Biology	83	89	1	AP Biology^a^	28	24
**2**	Integrated Biology	66	66	2	Biology	15	13
**3**	Bioethics	19	18	3	Biology	10	8
	**Group B (course)**	**No Pre-test (N)**	**Post-test (N)**		**Group B (course)**	**No Pre-test (N)**	**Post-test (N)**
**4**	Biology	0	40	4	Environ. Sciences^b^	0	7
**5**	Chemistry	0	49	5	Biology	0	15
**6**	Biology	0	61	6	Honors Biology	0	41

a Advanced Placement Biology.

b Environmental Sciences.

In order to assess the acquisition of higher-order justification skills, students used the summative assessment provided in our curriculum as the pre- and post-test. We designed the curriculum to scaffold students’ ability to write a persuasive bioethical position; by the time they participated in the assessment, Cohort 1 students had opportunities to discuss the elements of a strong justification as well as practice in analyzing case studies. For our research, both Cohort 1 and 2 students were asked to analyze the case study of “Ashley X” (Table S2), a young girl with a severe neurological impairment whose parents wished to limit her growth through a combination of interventions so that they could better care for her. Students were asked to respond to the ethical question: “Should one or more medical interventions be used to limit Ashley’s growth and physical maturation? If so, which interventions should be used and why?” In their answer, students were encouraged to develop a well-reasoned written position by responding to five questions that reflected elements of a strong justification. One difficulty in evaluating a multifaceted science-related learning task (analyzing a bioethical case study and justifying a position) is that a traditional multiple-choice assessment may not adequately reflect the subtlety and depth of student understanding. We used a rubric to assess student responses to each of the following questions (Q) on a scale of 1 to 4; these questions represent key elements of a strong justification for a bioethical argument:

- Q1: Student Position: What is your decision?
- Q2: Factual Support: What facts support your decision? Is there missing information that could be used to make a better decision?
- Q3: Interests and Views of Others: Who will be impacted by the decision and how will they be impacted?
- Q4: Ethical Considerations: What are the main ethical considerations?
- Q5: Evaluating Alternative Options: What are some strengths and weaknesses of alternate solutions?

In keeping with our focus on the process of reasoning rather than on having students draw any particular conclusion, we did not assess students on which position they took, but on how well they stated and justified the position they chose.

We used a rubric scoring guide to assess student learning, which aligned with the complex cognitive challenges posed by the task (Table S3). Assessing complex aspects of student learning is often difficult, especially evaluating how students represent their knowledge and competence in the domain of bioethical reasoning. Using a scoring rubric helped us more authentically score dimensions of students’ learning and their depth of thinking. An outside scorer who had previously participated in CURE workshops, has secondary science teaching experience, and who has a Masters degree in Bioethics blindly scored all student pre- and post-tests. Development of the rubric was an iterative process, refined after analyzing a subset of surveys. Once finalized, we confirmed the consistency and reliability of the rubric and grading process by re-testing a subset of student surveys randomly selected from all participating classes. The Cronbach alpha reliability result was 0.80 [19].

The rubric closely followed the framework introduced through the curricular materials and reinforced through other case study analyses. For example, under Q2, *Factual Support*, a student rated 4 out of 4 if their response demonstrated the following:

- The justification uses the relevant scientific reasons to support student’s answer to the ethical question.
- The student demonstrates a solid understanding of the context in which the case occurs, including a thoughtful description of important missing information.
- The student shows logical, organized thinking. Both facts supporting the decision and missing information are presented at levels exceeding standard (as described above).

An example of a student response that received the highest rating for Q2 asking for factual support is: “Her family has a history of breast cancer and fibrocystic breast disease. She is bed-bound and completely dependent on her parents. Since she is bed-bound, she has a higher risk of blood clots. She has the mentality of an infant. Her parents’ requests offer minimal side effects. With this disease, how long is she expected to live? If not very long then her parents don’t have to worry about growth. Are there alternative measures?”

In contrast, a student rated a 1 for responses that had the following characteristics:

- Factual information relevant to the case is incompletely described or is missing.
- Irrelevant information may be included and the student demonstrates some confusion.

An example of a student response that rated a 1 for Q2 is: “She is unconscious and doesn’t care what happens.”

All data were entered into SPSS (Statistical Package for the Social Sciences) and analyzed for means, standard deviations, and statistically significant differences. An Analysis of Variance (ANOVA) was used to test for significant overall differences between the two cohort groups. Pre-test and post-test composite scores were calculated for each student by adding individual scores for each item on the pre- and post-tests. The composite score on the post-test was identical in form and scoring to the composite score on the pre-test. The effect of the CURE treatment on post-test composite scores is referred to as the Main Effect, and was determined by comparing the post-test composite scores of the Cohort 1 (CURE) and Cohort 2 (Comparison) groups. In addition, Cohort 1 and Cohort 2 means scores for each test question (Questions 1–5) were compared within and between cohorts using t-tests.

#### CURE student perceptions of curriculum effect

During prior program evaluations, we asked teachers to identify what they believed to be the main impacts of bioethics instruction on students. From this earlier work, we identified several themes. These themes, listed below, were further tested in our current study by asking students in the treatment group to assess themselves in these five areas after participation in the lesson, using a retrospective pre-test design to measure self-reported changes in perceptions and abilities [20].

- Interest in the science content of class (before/after) participating in the Ethics unit.
- Ability to analyze issues related to science and society and make well-justified decisions (before/after) participating in the Ethics unit.
- Awareness of ethics and ethical issues (before/after) participating in the Ethics unit.
- Understanding of the connection between science and society (before/after) participating in the Ethics unit.
- Ability to listen to and discuss different viewpoints (before/after) participating in the Ethics unit.

After Cohort 1 (CURE) students participated in the Bioethics 101 curriculum, we asked them to indicate the extent to which they had changed in each of the theme areas we had identified using Likert-scale items on a retrospective pre-test design [21], with 1 =  None and 5 =  A lot!. We used paired t-tests to examine self-reported changes in their perceptions and abilities. The retrospective design avoids response-shift bias that results from overestimation or underestimation of change since both before and after information is collected at the same time [20].

## Results

### Student Demographics

Demographic information is provided in Table 3. Of those students who reported their gender, a larger number were female (N = 258) than male (N = 169), 60% and 40%, respectively, though female students represented a larger proportion of Cohort 1 than Cohort 2. Students ranged in age from 14 to 18 years old; the average age of the students in both cohorts was 15. Students were enrolled in a variety of science classes (mostly Biology or Honors Biology). Because NIH recognizes a difference between race and ethnicity, students were asked to respond to both demographic questions. Students in both cohorts were from a variety of ethnic and racial backgrounds.

**Table 3:** Demographic Characteristics of Cohort 1 (CURE Treatment) and Cohort 2 (Comparison) Students^a^.

		Cohort 1(%)	Cohort 2 (%)
**Sex**	**Female**	202 (63)	56 (52)
	**Male**	118 (37)	51 (48)
**Age**	**14**	29 (9.1)	33 (30.0)
	**15**	187 (58.4)	32 (29.1)
	**16**	63 (19.7)	14 (12.7)
	**17**	27 (8.5)	14 (12.7)
	**18**	12 (3.8)	17 (15.5)
**Race**	**Amer. Indian/AL Native** b	3 (0.9)	1 (0.9)
	**Asian**	14 (4.4)	14 (12.8)
	**Black/African American**	8 (2.5)	4 (3.7)
	**Native HA** c **or Pacific Islander**	2 (0.6)	1 (0.9)
	**White**	260 (81.3)	48 (44)
	**More than One Race**	22 (6.9)	15 (13.8)
	**Other**	9 (2.8)	24 (22)
**Ethnicity**	**Hispanic or Latino**	20 (6.3)	29 (27)
	**Not Hispanic or Latino**	295 (92.2)	78 (73)

a Percentages of individual items might not equal 100% because of missing responses.

b American Indian/Alaska Native.

c Native Hawaiian.

### Pre- and Post-Test Results for CURE and Comparison Students

Post-test composite means for each cohort (1 and 2) and group (A and B) are shown in Table 4. Students receiving CURE instruction earned significantly higher (p<0.001) composite mean scores than students in comparison classrooms. Cohort 1 (CURE) students (N = 323) post-test composite means were 10.73, while Cohort 2 (Comparison) students (N = 108) had post-test composite means of 9.16. The ANOVA results (Table 5) showed significant differences in the ability to craft strong justifications between Cohort 1 (CURE) and Cohort 2 (Comparison) students *F* (1, 429) = 26.64, p<0.001.

**Table 4:** Cohort Group Comparison of Post-Test Composite Mean Scores.

		Descriptive Statistics		
		Mean	SD^a^	N
**Cohort 1**	**Group A**	10.72	2.63	173
	**Group B**	10.75	2.77	150
	**Total**	10.73	2.70	323
**Cohort 2**	**Group A**	9.96	3.48	45
	**Group B**	8.59	2.20	63
	**Total**	9.16	2.88	108

a Standard Deviation.

**Table 5:** Analysis of Variance for Cohort Main Effects.

Source	Sum of Squares	Degrees of Freedom	Mean Squares	F	Significance
**Cohort Differences** **(between groups)**	200.33	1	200.33	26.64	<0.001
**Subject Interaction** **(within groups)**	3225.89	429	7.52		
**Total**	3426.22	430			

We also examined if the pre-test had a priming effect on the students’ scores because it provides an opportunity to practice or think about the content. The pre-test would not have this effect on the comparison group because they were not exposed to CURE teaching or materials. If the pre-test provides a practice or priming effect, this would result in higher post-test performance by CURE students receiving the pre-test than by CURE students not receiving the pre-test. For this comparison, the *F* (1, 321) = 0.10, p = 0.92. This result suggests that the differences between the CURE and comparison groups are attributable to the treatment condition and not a priming effect of the pre-test.

**Figure 1:**
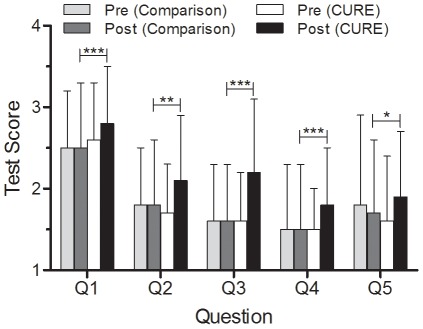
Cohort 1 (CURE) and Cohort 2 (Comparison) Pre- and Post-Test Scores (N = 431). Mean scores for individual items of the pre-test for each cohort revealed no differences between groups for any of the items (Cohort 1, CURE, N = 323; Cohort 2, Comparison, N = 108). Post-test gains of Cohort 1 (CURE) relative to Cohort 2 (Comparison) were statistically significant for all questions. (Question (Q) 1) What is your decision? (Q2) What facts support your decision? Is there missing information that could be used to make a better decision? (Q3) Who will be impacted by the decision and how will they be impacted? (Q4) What are the main ethical considerations? and (Q5)What are some strengths and weaknesses of alternate solutions? Specifically: (Q1), (Q3), (Q4) were significant at p<0.001 (***); (Q2) was significant at p<0.01 (**); and (Q5) was significant at p<0.05 (*). Lines represent standard deviations.

After differences in main effects were investigated, we analyzed differences between and within cohorts on individual items (Questions 1–5) using t-tests. The Mean scores of individual questions for each cohort are shown in Figure 1. There were no significant differences between Cohort 1 (CURE) and Cohort 2 (Comparison) on pre-test scores. In fact, for Q5, the mean pre-test scores for the Cohort 2 (Comparison) group were slightly higher (1.8) than the Cohort 1 (CURE) group (1.6). On the post-test, the Cohort 1 (CURE) students significantly outscored the Cohort 2 (Comparison) students on all questions; Q1, Q3, and Q4 were significant at p<0.001, Q2 was significant at p<0.01, and Q5 was significant at p<0.05. The largest post-test difference between Cohort 1 (CURE) students and Cohort 2 (Comparison) students was for Q3, with an increase of 0.6; all the other questions showed changes of 0.3 or less. Comparing Cohort 1 (CURE) post-test performance on individual questions yields the following results: scores were highest for Q1 (mean = 2.8), followed by Q3 (mean = 2.2), Q2 (mean = 2.1), and Q5 (mean = 1.9). Lowest Cohort 1 (CURE) post-test scores were associated with Q4 (mean = 1.8).

Overall, across all four groups, mean scores for Q1 were highest (2.6), while scores for Q4 were lowest (1.6). When comparing within-Cohort scores on the pre-test versus post-test, Cohort 2 (Comparison Group) showed little to no change, while CURE students improved on all test questions.

### CURE Student Perceptions of Curriculum Effect

**Figure 2:**
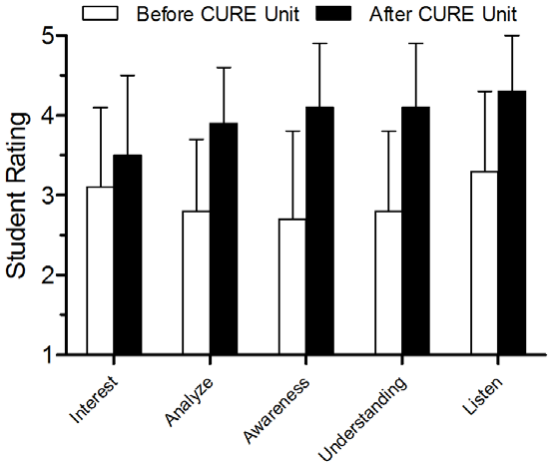
Student Perceptions about Participation in the CURE Ethics Unit. Mean scores for individual items of the retrospective items on the post-test for Cohort 1 students revealed significant gains (p<0.001) in all self-reported items: **Interest** in science (N = 308), ability to **Analyze** issues related to science and society and make well-justified decisions (N = 306), **Awareness** of ethics and ethical issues (N = 309), **Understanding** of the connection between science and society (N = 308), and the ability to **Listen** and discuss different viewpoints (N = 308). Lines represent standard deviations.

After using our resources, Cohort 1 (CURE) students showed highly significant gains (p<0.001) in all areas examined: interest in science content, ability to analyze socio-scientific issues and make well-justified decisions, awareness of ethical issues, understanding of the connection between science and society, and the ability to listen to and discuss viewpoints different from their own (Figure 2). Overall, students gave the highest score to their ability to listen to and discuss viewpoints different than their own after participating in the CURE unit (mean = 4.2). Also highly rated were the changes in understanding of the connection between science and society (mean = 4.1) and the awareness of ethical issues (mean = 4.1); these two perceptions also showed the largest change pre-post (from 2.8 to 4.1 and 2.7 to 4.1, respectively).

## Discussion

NWABR’s teaching materials provide support both for general ethics and bioethics education, as well as for specific topics such as embryonic stem cell research. These resources were developed to provide teachers with classroom strategies, ethics background, and decision-making frameworks. Teachers are then prepared to share their understanding with their students, and to support their students in using analysis tools and participating in effective classroom discussions. Our current research grew out of a desire to measure the effectiveness of our professional development and teaching resources in fostering student ability to analyze a complex bioethical case study and to justify their positions.

Consistent with the findings of SSI researchers and our own prior anecdotal observations of teacher classrooms and student work, we found that students improve in their analytical skill when provided with reasoning frameworks and background in concepts such as beneficence, respect, and justice. Our research demonstrates that structured reasoning approaches can be effectively taught at the secondary level and that they can improve student thinking skills. After teachers participated in a two-week professional development workshop and utilized our Bioethics 101 curriculum, within a relatively short time period (five lessons spanning approximately one to two weeks), students grew significantly in their ability to analyze a complex case and justify their position compared to students not exposed to the program. Often, biology texts present a controversial issue and ask students to “justify their position,” but teachers have shared with us that students frequently do not understand what makes a position or argument well-justified. By providing students with opportunities to evaluate sample justifications, and by explicitly introducing a set of elements that students should include in their justifications, we have facilitated the development of this important cognitive skill.

The first part of our research examined the impact of CURE instruction on students’ ability to analyze a case study. Although students grew significantly in all areas, the highest scores for the Cohort 1 (CURE) students were found in response to Q1 of the case analysis, which asked them to clearly state their own position, and represented a relatively easy cognitive task. This question also received the highest score in the comparison group. Not surprisingly, students struggled most with Q4 and Q5, which asked for the ethical considerations and the strengths and weaknesses of different solutions, respectively, and which tested specialized knowledge and sophisticated analytical skills. The area in which we saw the most growth in Cohort 1 (CURE) (both in comparison to the pre-test and in relation to the comparison group) was in students’ ability to identify stakeholders in a case and state how they might be impacted by a decision (Q3). Teachers have shared with us that secondary students are often focused on their own needs and perspectives; stepping into the perspectives of others helps enlarge their understanding of the many views that can be brought to bear upon a socio-scientific issue.

Many of our teachers go far beyond these introductory lessons, revisiting key concepts throughout the year as new topics are presented in the media or as new curricular connections arise. Although we have observed this phenomenon for many years, it has been difficult to evaluate these types of interventions, as so many teachers implement the concepts and ideas differently in response to their unique needs. Some teachers have used the Bioethics 101 curriculum as a means for setting the tone and norms for the entire year in their classes and fostering an atmosphere of respectful discussion. These teachers note that the “opportunity cost” of investing time in teaching basic bioethical concepts, decision-making strategies, and justification frameworks pays off over the long run. Students’ understanding of many different science topics is enhanced by their ability to analyze issues related to science and society and make well-justified decisions. Throughout their courses, teachers are able to refer back to the core ideas introduced in Bioethics 101, reinforcing the wide utility of the curriculum.

The second part of our research focused on changes in students’ self-reported attitudes and perceptions as a result of CURE instruction. Obtaining accurate and meaningful data to assess student self-reported perceptions can be difficult, especially when a program is distributed across multiple schools. The traditional use of the pretest-posttest design assumes that students are using the same internal standard to judge attitudes or perceptions. Considerable empirical evidence suggests that program effects based on pre-posttest self-reports are masked because people either overestimate or underestimate their pre-program perceptions [20], [22]–[26]. Moore and Tananis [27] report that response shift can occur in educational programs, especially when they are designed to increase students’ awareness of a specific construct that is being measured. The retrospective pre-test design (RPT), which was used in this study, has gained increasing prominence as a convenient and valid method for measuring self-reported change. RPT has been shown to reduce response shift bias, providing more accurate assessment of actual effect. The retrospective design avoids response-shift bias that results from overestimation or underestimation of change since both before and after information is collected at the same time [20]. It is also convenient to implement, provides comparison data, and may be more appropriate in some situations [26]. Using student self-reported measures concerning perceptions and attitudes is also a meta-cognitive strategy that allows students to think about their learning and justify where they believe they are at the end of a project or curriculum compared to where they were at the beginning.

Our approach resulted in a significant increase in students’ own perceived growth in several areas related to awareness, understanding, and interest in science. Our finding that student interest in science can be significantly increased through a case-study based bioethics curriculum has implications for instruction. Incorporating ethical dilemmas into the classroom is one strategy for increasing student motivation and engagement with science content. Students noted the greatest changes in their own awareness of ethical issues and in understanding the connection between science and society. Students gave the highest overall rating to their ability to listen to and discuss viewpoints different from their own after participation in the bioethics unit. This finding also has implications for our future citizenry; in an increasingly diverse and globalized society, students need to be able to engage in civil and rational dialogue with others who may not share their views.

Conducting research studies about ethical learning in secondary schools is challenging; recruiting teachers for Cohort 2 and obtaining consent from students, parents, and teachers for participation was particularly difficult, and many teachers faced restraints from district regulations about curriculum content. Additional studies are needed to clarify the extent to which our curricular materials alone, without accompanying teacher professional development, can improve student reasoning skills.

Teacher pre-service training programs rarely incorporate discussion of how to address ethical issues in science with prospective educators. Likewise, with some noticeable exceptions, such as the work of the University of Pennsylvania High School Bioethics Project, the Genetic Science Learning Center at the University of Utah, and the Kennedy Institute of Ethics at Georgetown University, relatively few resources exist for high school curricular materials in this area. Teachers have shared with us that they know that such issues are important and engaging for students, but they do not have the experience in either ethical theory or in managing classroom discussion to feel comfortable teaching bioethics topics. After participating in our workshops or using our teaching materials, teachers shared that they are better prepared to address such issues with their students, and that students are more engaged in science topics and are better able to see the real-world context of what they are learning.

Preparing students for a future in which they have access to personalized genetic information, or need to vote on proposals for stem cell research funding, necessitates providing them with the tools required to reason through a complex decision containing both scientific and ethical components. Students begin to realize that, although there may not be an absolute “right” or “wrong” decision to be made on an ethical issue, neither is ethics purely relative (“my opinion versus yours”). They come to realize that all arguments are not equal; there are stronger and weaker justifications for positions. Strong justifications are built upon accurate scientific information and solid analysis of ethical and contextual considerations. An informed citizenry that can engage in reasoned dialogue about the role science should play in society is critical to ensure the continued vitality of the scientific enterprise.

*“I now bring up ethical issues regularly with my students, and use them to help students see how the concepts they are learning apply to their lives…I am seeing positive results from my students, who are more clearly able to see how abstract science concepts apply to them.”*

*– CURE Teacher*

*“In ethics, I’ve learned to start thinking about the bigger picture. Before, I based my decisions on how they would affect me. Also, I made decisions depending on my personal opinions, sometimes ignoring the facts and just going with what I thought was best. Now, I know that to make an important choice, you have to consider the other people involved, not just yourself, and take all information and facts into account.”*

*– CURE Student*

## Supporting Information

Table S1 Bioethics 101 Lesson Overview.
(DOCX)

Table S2 Case Study for Assessment.
(DOCX)

Table S3 Grading Rubric for Pre- and Post-Test: Ashley’s Case.
(DOCX)
