# Supplementary material for: Fostering Critical Thinking, Reasoning, and Argumentation Skills through Bioethics Education
Source: PLoS One. 2012 May 11;7(5):e36791. doi: 10.1371/journal.pone.0036791 (PMC3350470; doi:10.1371/journal.pone.0036791)
Supplement: Table S1 — Bioethics 101 Lesson Overview. (DOCX) [file pone.0036791.s001.docx]

Table S1. Bioethics 101 Lesson Overview.

| **Lesson 1—Introduction to Bioethics** |
| --- |
| Students are introduced to the characteristics of an ethical question and learn to distinguish ethical questions from other types of questions, such as legal or scientific questions. Student then identify an ethical question and participate in an ethical dilemma involving the distribution of a scarce resource—a flu vaccine—during a flu outbreak. Students are asked to determine the best course of action in the face of conflicting choices, while examining the underlying themes that serve as a basis for their reasoning. Lastly, students experience how relevant facts influence decision-making. |
| **Lesson 2—Principles of Bioethics** |
| Students consider questions with answers based on fact, preference, or reasoned judgment, and determine where those questions fall along the range of purely subjective to purely objective. Students then improvise short skits to illustrate familiar concepts such as fairness, respect, and ‘doing good’ which sets the foundation for the Principles of Bioethics: Respect for Persons, Maximize Benefits/Minimize Harms, and Justice. Students then apply these bioethical principles to the Pandemic Flu ethical dilemma they were introduced to in Lesson One. |
| **Lesson 3—Finding the Stakeholders** |
| Students read a case study about Dennis, a 14-year old boy who has been diagnosed with leukemia. The doctors treat the leukemia with chemotherapy, which dramatically reduces the number of Dennis’s blood cells; Dennis, however, refuses life-saving blood transfusions because they conflict with his faith. Students identify an ethical question to explore, and consider how the Principles of Bioethics (Respect for Persons, Maximizing Benefits/Minimizing Harms, and Justice) relate to the case. Students then identify the stakeholders—the people or institutions that are affected by the outcome—and work in small groups to clarify stakeholder values, interests, and concerns. After stakeholder groups present their positions to the class, the class generates options for possible resolutions to the case. |
| **Lesson 4—Making a Strong Justification** |
| In this lesson, students learn the characteristics of a strong justification and apply them to a decision about an ethical question. Students brainstorm what makes a weak justification, and are then primed to identify what makes a strong justification through their participation in a silent debate. Students refer to the Case Study: Dennis’s Decision from Lesson 3, and evaluate a number of pre-written justifications for that case. For each justification, students consider whether or not a decision has been made, if scientific facts have been included, if stakeholder views are represented, if there is reference to bioethical principles, and if alternate solutions are considered. Once students understand the elements of a well-crafted justification, they come to their own decision about Dennis and write their own justification. |
| **Lesson 5—Putting it all Together** |
| In this lesson, students consider the case of a young doctor hired by a U.S. pharmaceutical company to test a new antibiotic in Nigeria during a meningitis epidemic. Students work through a Decision-Making Framework in small groups, in which they identify the ethical question, determine which facts are known or unknown, consider the values of different stakeholder groups, generate possible solutions, and then make and justify a decision about the case. This is a jigsaw exercise, in which students first meet in ‘like’ stakeholder groups to become experts in the values and concerns of that group. Teams are then rearranged so that each new group has students from different stakeholder viewpoints. After sharing the views and values of each stakeholder group with their peers, groups work together to generate options for solutions to the case study. Lastly, students come to individual decisions about the case and write a thorough justification. |
