# Supplementary material for: Fostering Critical Thinking, Reasoning, and Argumentation Skills through Bioethics Education
Source: PLoS One. 2012 May 11;7(5):e36791. doi: 10.1371/journal.pone.0036791 (PMC3350470; doi:10.1371/journal.pone.0036791)
Supplement: Table S2 — Case Study for Assessment. (DOCX) [file pone.0036791.s002.docx]

Table S2. Case Study for Assessment.

| **Ashley’s Case** |
| --- |
| *Case study originally developed by Jacob Dahlke* |
| Ashley, at age 6½, could not roll over, sit up or hold her head up, or use language. Developmentally, she was like an infant. Ashley’s parents, who have two other healthy children, had cared for Ashley in their home since birth. Ashley was diagnosed with “static encephalopathy,” meaning that her brain had stopped developing. Doctors determined that there was no chance of Ashley improving over time. |
| Ashley’s parents grew concerned over their abilities to continue to care for Ashley at home. With her continued growth and development, she would eventually become too large for them to manage her needs, including feeding her, changing her, bathing her, and positioning her during the night. Additionally, they were concerned at the prospects of her sexual development, including menstruation, breast development, and her fertility. |
| Ashley’s parents made three requests of doctors at Children’s Hospital and Regional Medical Center in Seattle, Washington. First, they wanted Ashley to have a hysterectomy (removal of her uterus) in order to prevent any risk of menstruation and/or pregnancy. Although there are methods like birth control pills to address these issues, they are accompanied by the possibility of long term side effects. One risk, blood clots, is considerable in a patient who is bed bound and unable to move herself. Second, they requested the removal of her breast buds, which would eliminate the development of breasts altogether. Ashley’s parents argued that her breasts would cause discomfort with the straps used to hold her in her chair, and that breast discomfort was a known problem for some adult women in the family. There was also a family history of fibrocystic breast disease and breast cancer. Without breasts, Ashley would be spared future mammograms and possible biopsies. Finally, Ashley’s parents requested medical treatment to limit her final adult height and weight through hormone therapy. High dose hormone therapy to limit height was a common treatment for “tall girls” in the 1960’s and 70’s and the medical risks over the long term are known to be limited. |
| The ethics committee noted that there was great need for caution for such a procedure, as there have been many documented cases of past abuses of people with physical and developmental disabilities. Dr. Doug Diekema (who, with Dr. Daniel Gunther, published their paper on Ashley in the *Archives of Pediatric and Adolescent Medicine*) acted as ethicist on this case, and was part of the group that decided the outcome of the parent’s requests. Dr. Diekema noted that there were few medical risks involved with the hysterectomy and removal of breast buds (standard surgical procedural risks), and only slightly higher risks associated with the hormone therapy (such as blood clotting). |
| Critics noted that this combination of surgery and hormones to prevent a person from maturing into an adult was unprecedented in medical history. There were also worries about Ashley’s rights as a patient, as her parents were making this decision without her ability to contribute. There was a general debate about the potential ‘slippery slope’ of adapting the bodies of the disabled to suit the needs of the caregivers, unless it could be justified that this change was also in the patient’s (Ashley’s) best interests. An ethics consultation involving about 20 individuals was performed prior to making the decision. The consultation included a developmental specialist, Ashley’s primary care provider, and her hormone specialist. Although Ashley’s parents attended the consultation, they were not a part of the deliberation. |
