# Supplementary material for: Fostering Critical Thinking, Reasoning, and Argumentation Skills through Bioethics Education
Source: PLoS One. 2012 May 11;7(5):e36791. doi: 10.1371/journal.pone.0036791 (PMC3350470; doi:10.1371/journal.pone.0036791)
Supplement: Table S3 — Grading Rubric for Pre- and Post-Test: Ashley’s Case. (DOCX) [file pone.0036791.s003.docx]

Table S3. Grading Rubric for Pre- and Post-Test: Ashley’s Case.

| **Ethical Question**: Should one or more medical interventions be used to limit Ashley’s growth and physical maturation? If so, which interventions should be used and why? | | | |
| --- | --- | --- | --- |
| **Exemplary-4** | **Proficient-3** | **Partially Proficient-2** | **Developing-1** |
| **1. What is your decision? Why is that the best option? (A position that relates directly to the ethical question has been clearly stated and explained.)** | | | |
| Student states the best option and discusses all of the interventions with pros/cons, or student states the best option and uses ethical principles to support decision. Student shows thoughtful consideration and organized thinking. Student uses accurate information to support his/her decision. | The student’s choice of best option is clearly stated, but may not mention all options. Student shows clear thinking. -OR- Student states the best option, and provides accurate information to support his/her decision, or student discusses other interventions. | Student does not clearly state the best option or does not state the best option as what should be done (ex. “If I were Ashley, I would want the procedures,” or “The procedures seem unnecessary,”). Student does not give any reasons to support his/her decision. | Student states an option that is not one of the options for the case (ex. assisted suicide) or student response shows no understanding of the situation or the question being asked. |
| **2. What facts support your decision? Is there missing information that could be used to make a better decision? (The facts and science content can be confirmed or refuted regardless of personal or cultural views.)** | | | |
| The justification *uses the relevant scientific reasons* to support student’s answer to the ethical question. Student demonstrates a solid understanding of the context in which the case occurs, including a thoughtful description of important missing information. Student shows logical, organized thinking. Both facts supporting the decision and missing information are presented at levels exceeding standard (as described above). | The main relevant facts are identified. All scientific concepts are correctly presented. Student shows clear thinking. Information that is missing from the case that would influence decision-making is referenced. Both facts supporting the decision and missing information are presented at levels meeting standard (as described above). | Factual information relevant to the case is described but some key facts may be missing and some irrelevant information may also be included. Information that is missing from the case that would influence decision-making may be lacking. -OR- Student presents only facts *or* missing information. | Factual information relevant to the case is incompletely described or is missing. Irrelevant information may be included and student demonstrates some confusion. |
| **3. Who will be impacted by the decision and how will they be impacted? (There are a variety of views and interests in the decision, and more than one individual or group will be affected by the outcome.)** | | | |
| Three or more stakeholders, the ways in which they are impacted, and their values, interests, and/or concerns are identified or 4 or more stakeholders and the ways in which they are impacted are identified. | Three stakeholders and the ways in which they are impacted are identified or 4 stakeholders are identified without mention of the impacts on them. | Two stakeholders and the ways in which they are impacted are identified or 3 stakeholders are identified without mention of the impacts on them. | Only 1 stakeholder and the way in which this stakeholder is impacted is identified or 2 stakeholders are identified without mention of the impacts on them. |
| **4. What are the main ethical considerations? (Ethical considerations may include Respect for Persons, Maximize Benefits/Minimize Harms, and Justice.)** | | | |
| The student *evaluates the case in depth using one or more ethical considerations.* The student shows exceptional understanding of how one or more ethical considerations relates to the case. The student’s decision is supported by the thorough, thoughtful application of the consideration(s) to the case. The student demonstrates organized thinking, and his/her conclusions flow logically from premises. Student response includes analysis/evaluation of the case with regards to issues of consent, best interest, and/or benefits/harms. | The student demonstrates an understanding of the ethical consideration(s) related to the case. The student provides clear explanation of how ethical considerations support his/her decision. Student response includes issues of consent, best interest, and/or benefits/harms. | The student demonstrates a general awareness ethical considerations and how they relate to the case, but may not articulate the relationship clearly or provide enough explanation. The student demonstrates mostly clear and organized thinking, but portions of the answer may be unclear, disorganized, or incomplete. Student response seems to refer to issues of consent, best interest, and/or benefits/harms. | The student lacks an awareness of ethical principles or does not properly relate them to the case. The student demonstrates some confused or disorganized thinking. Student response does not include ethical considerations (ex. legal considerations). |
| **5. What are the strengths and weaknesses of alternate solutions? (No one decision will satisfy all parties. A thorough justification considers various positions.)** | | | |
| Thorough analysis of the alternate solutions that includes multiple strengths and weaknesses and/or multiple alternate solutions. The writing is clear and organized. | Presents both the strengths and the weaknesses of the alternate solution(s). | Only discusses the strengths or the weaknesses of the alternate solution or contains either misconceptions or unrealistic strengths or weaknesses (ex. Ashley’s brain will start to develop or being able to maturate normally is a strength for her) | No alternate solutions are discussed, or presents strengths and/or weaknesses for solution, not alternate solutions, or presents unrealistic alternatives (ex. assisted suicide). |
